# Lésions cérébrales hémorragiques multiples révélatrices d’un mélanome métastatique

**DOI:** 10.11604/pamj.2019.33.78.16077

**Published:** 2019-06-03

**Authors:** Lala Andriamasinavalona Rajaonarison, Ratsitohara Santatra Razafindrasata

**Affiliations:** 1 Faculté de Médecine d’Antananarivo, CHU Joseph Raseta Befelatanana, Service de Neurologie, Antananarivo, Madagascar

**Keywords:** Crises épileptiques, mélanome, Epileptic seizures, melanoma

## Abstract

Nearly 75% of patients with metastatic melanoma develop brain metastases. We here report the case of an 83 year-old woman hospitalized for secondarily generalized clonic seizures of the left leg with partial convulsive seizures in the Resuscitation Department. Melanoma resection of the left ankle had been performed 6 months before her admission. Neurological examination showed left ataxic crural monoparesis. Electro-encephalogram showed central and right frontal focus with left-sided dissemination. Gadolinium-enhanced magnetic resonance imaging (MRI) of the brain showed multiple supratentorial and subtentorial encephalic lesions with varying size and shape, with T1 hypersignal (A and A’), haemorrhage on T2*-weighted sequences (B and B’), gadolinium-enhancing T1 with perilesional edema on Flair sequences. Positron emission tomography (PET) showed multiple lymph node and bone metastases. Lymph node biopsy was negative for VE1 antibody with no BRAFV600E mutation by immunohistochemistry. An increase in the number of metastatic lesions was observed during control brain CT scan despite 10 brain radiotherapy sessions motivating palliative care. Epileptic seizures were controlled with levetiracetam. In patient with multiple hemorrhagic and spontaneous brain lesions, it is essential to obtain informations on patient’s history of melanoma and to perform a thorough dermatologic examination in order to investigate its cause and to establish adequate therapeutic treatment.

## Image en médecine

Près de 75% des patients avec un mélanome métastatique développent des métastases cérébrales au cours de leurs pathologies. Nous rapportons le cas d'une femme de 83 ans hospitalisée pour des crises cloniques de la jambe gauche secondairement généralisée, en état de mal épileptique convulsif partiel motivant une prise en charge auprès du service de réanimation. Une exérèse d'un mélanome de la cheville gauche était pratiquée 6 mois avant son admission. L'examen neurologique montrait une mono parésie ataxique crurale gauche. L'électro-encéphalogramme montrait un foyer central et frontal droit avec diffusion à gauche. L'imagerie par résonance magnétique (IRM) encéphalique avec injection de gadolinium a montré des lésions encéphaliques multiples de taille et de forme différentes, en sus et sous tentoriel, en hyper signal T1 (A et A'), hémorragique en T2*(B et B'), prenant le gadolinium en T1 gado avec œdème péri lésionnel en Flair. La tomographie par émission de positon a montré de multiples métastases ganglionnaires et osseuses. La recherche du clone VE-1 était négative après biopsie ganglionnaire avec absence de mutation BRAFV600E à l'immunohistochimie. Une augmentation du nombre des lésions métastatiques a été notée lors d'un scanner cérébral de contrôle malgré une radiothérapie cérébrale sur 10 séances motivant la mise sous soins palliatif. Les crises épileptiques ont été contrôlées sous lévétiracétam. Devant des lésions cérébrales multiples, hémorragiques et spontanées, il est important de rechercher la notion de mélanome dans l'antécédent du patient et de faire un examen dermatologique minutieux. Ceci pour l'enquête étiologique et conditionnant la prise en charge thérapeutique du patient.

**Figure 1:**
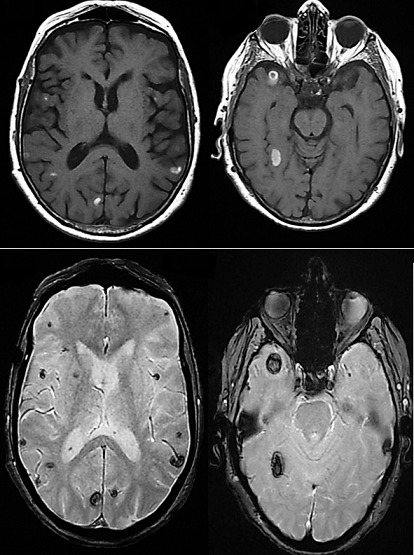
IRM encéphalique: hypersignal spontané multifocal de taille et de forme différentes T1 (A et A’), de nature hémorragique en T2 (B et B’)

